# Custom-made dynamic 3-dimensional−printed prostheses for chest wall reconstruction: A multicenter study

**DOI:** 10.1016/j.xjtc.2025.09.010

**Published:** 2025-09-19

**Authors:** Jose Ramón Cano, Unai Jiménez, Juan Carlos Trujillo, Jose M. Galbis, Donato Monopoli, David Pérez, Sara Fra-Fernández, Naia Uribe-Etxebarria, Elisabeth Martínez, Miriam Estors, Belinda Mentado, Gemma María Muñoz-Molina, Ricardo Medina, Monica Lorenzo, Jorge Hernandez-Ferrandez, Wolker Tavárez, Michelle Leung, Nicolás Moreno-Mata

**Affiliations:** a Thoracic Surgery Department, Complejo Hospitalario Universitario Insular Materno Infantil, Las Palmas de Gran Canaria, Spain; b Thoracic Surgery Department, Hospital Universitario Cruces, Barakaldo, Spain; c Thoracic Surgery Department, Hospital de la Santa Creu i Sant Pau, Barcelona, Spain; d Thoracic Surgery Department, Hospital Universitario de La Ribera, Valencia, Spain; e Department of Biomedical Engineering, Instituto Tecnológico de Canarias, Las Palmas de Gran Canaria, Spain; f Thoracic Surgery Department, Hospital Ramon y Cajal, Madrid, Spain

**Keywords:** chest wall reconstruction, custom-made prosthesis, dynamic implant, sternocostal resection, 3D printing, thoracic surgery, patient-specific implant

## Abstract

**Objective:**

To assess the technical feasibility and surgical safety of dynamic, 3-dimensional (3D) customized prostheses for complex chest wall reconstruction across a multicenter experience.

**Methods:**

We conducted a retrospective, descriptive observational study involving 51 patients from 5 hospitals in Spain who underwent chest wall reconstruction via a custom-designed, spring-like 3D-printed titanium implants between 2016 and 2023. Data collected included surgical indication (oncologic, traumatic, or infectious), patient demographics, prosthesis type, operative time, hospital stay, complications, and follow-up outcomes.

**Results:**

Indications included 35 oncologic, 11 traumatic, 3 infectious, and 2 functional cases (winged scapula). A total of 51 prostheses were implanted, including bilateral and unilateral sternocostal, costal, costovertebral, sternoclavicular, and scapulothoracic reconstructions. The mean operative time was 270 minutes (range, 75-720 minutes), and median follow-up was 2.5 years (range, 3 months to 8 years). Four minor complications were recorded, none requiring implant removal. No cases of prosthesis failure occurred independently of patient death.

**Conclusions:**

Dynamic 3D-customized prostheses provide a structurally stable yet flexible alternative to conventional rigid implants for chest wall reconstruction. Their spring-like geometry allows anatomical adaptation and facilitates intraoperative placement, contributing to safe and reproducible reconstruction in selected cases, with favorable medium-term outcomes. This multicenter experience suggests they may be a feasible and safe option in anatomically complex or functionally demanding thoracic reconstructions.

## Graphical Abstract

**Figure:**
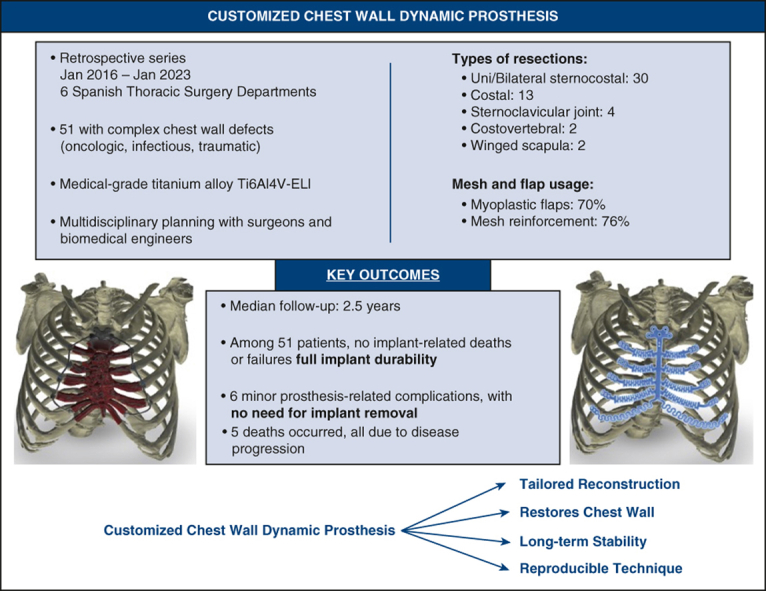

**Figure:**
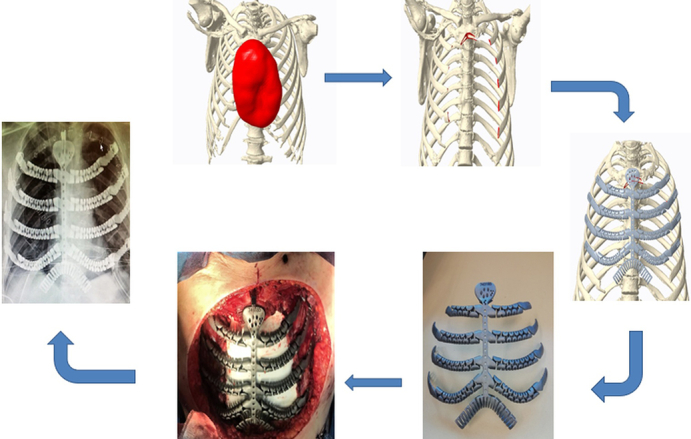
Redefining chest wall reconstruction with dynamic 3D implants: structure meets function.

**Central Message**

Dynamic 3D-printed implants may offer a promising surgical solution for complex chest wall defects, combining structural stability with preserved ventilatory mechanics.

**Perspective**

This study presents the largest multicenter experience using dynamic 3D-customized chest wall prostheses. These implants allow anatomically precise, functionally dynamic reconstructions, offering a standardized and reproducible alternative for complex thoracic defects with excellent medium-term outcomes.

Chest wall reconstruction remains one of the most complex challenges in thoracic surgery, particularly in cases involving large anterior defects, the sternum, or costosternal and costovertebral joints. Despite the availability of multiple techniques and materials, no clear consensus has been reached regarding the optimal approach, and reconstructive strategies continue to be highly heterogeneous.

Rigid materials—such as titanium implants, acrylic resins, or cryopreserved allografts—restore structural integrity but frequently restrict thoracic motion, potentially contributing to complications such as pain, paradoxical breathing, and cosmetic deformity. Moreover, these approaches carry a non-negligible risk of prosthetic migration, dislocation, or infection. In the absence of universally accepted solutions, most reconstructions are adapted to the available implant, rather than the implant being tailored to the patient's anatomy.^1^

Since 2014, advances in 3-dimensional (3D) printing have enabled the development of customized prostheses designed through preoperative collaboration with biomedical engineers.^2^, ^3^, ^4^, ^5^ These devices have improved anatomical accuracy and preoperative planning, yet they remain static and do not address the dynamic nature of the thoracic cage.

In 2016, the Thoracic Surgery Department at Complejo Hospitalario Universitario Insular Materno Infantil in Las Palmas de Gran Canaria, in collaboration with the Instituto Tecnológico de Canarias (ITC), developed a dynamic, patient-specific 3D-printed prosthesis intended to replicate both structural and biomechanical aspects of the chest wall.^6^ This design was progressively adopted by other centers in Spain for indications including anterior chest wall tumors, posttraumatic hernias, and scapular winging.

The aim of this study is to present the technical feasibility, surgical safety, and multicenter reproducibility of this novel dynamic prosthetic system. Rather than evaluating functional outcomes, this work focuses on the practical aspects of implementation, fixation strategies, and complication profiles on the basis of a series of patients treated across 5 institutions in Spain.

## Methods

This retrospective, descriptive observational study evaluated the use of dynamic, 3D-customized prostheses for complex chest wall reconstruction, conducted between April 2016 and December 2023 across 5 hospitals in Spain. These are high-volume, highly specialized centers, each performing approximately 15 to 20 chest wall resections annually.

Patients were considered candidates for dynamic custom-made prostheses if they presented with large chest wall defects—defined as resection involving ≥2 ribs on the anterior or posterolateral chest wall—or if the sternum, sternoclavicular, or costovertebral joints were affected. Traumatic indications included posttraumatic chest wall hernias with abnormal rib consolidation and winged scapula attributable to long thoracic nerve injury. Dynamic prostheses were selected in cases in which standard rigid systems were unlikely to achieve adequate anatomical and functional outcomes. All indications were confirmed through multidisciplinary evaluation.

Each surgical team operated independently at its respective institution. Given the novelty of the technology, the cohort comprised more than 50 cases from the 5 most experienced centers in Spain. The follow-up, with a minimum duration of 6 months, was conducted by the same surgical teams that performed the procedure.

The study was approved by the Ethics Committees of Hospital Universitario Ramón y Cajal, Madrid, Spain (institutional review board: 301/23, approval date: January 2024) and Complejo Hospitalario Universitario Insular Materno Infantil, Las Palmas, Spain (institutional review board: 2024-089-1, approval date: March 2024). The data underlying this article cannot be shared publicly because of the privacy of individuals that participated in the study. The data will be shared on reasonable request to the corresponding author.

In all cases, preoperative planning included a computed tomography scan with 3D bone reconstruction of the area to be resected—whether a tumor (with 2.5-cm margins), hernia, or scapular anomaly. A patient-specific prosthesis was designed in collaboration with biomedical engineers and subsequently manufactured using 3D printing technology.

The implants were designed by the ITC in collaboration with Osteobionix using parametric computer-aided design software (CREO, developed by PTC Inc). Designs were exported in STL (Standard Tessellation Language) format and manufactured by metal powder bed fusion using titanium alloy with aluminum and vanadium (extra low interstitials) (Ti6Al4V-ELI) powder, through either electron beam melting (EBM) or selective laser melting. Ti6Al4V-ELI is a medical-grade titanium alloy composed of titanium, aluminum (6%), and vanadium (4%), known for its high strength, corrosion resistance, and excellent biocompatibility with bone tissue. Postprocessing included removal of residual powder via Ti6Al4V-ELI sandblasting, manual support removal, polishing, and final surface treatment with corundum particles. The implants underwent 2 ultrasonic cleaning cycles: the first in distilled water with surgical-grade soap at 80°C for 40 minutes and the second in pure distilled water for 15 minutes.

Ti6Al4V EBM implants demonstrated a >97% reduction in bacterial adhesion and complete inhibition of biofilm formation. The zwitterionic surface further exhibited biocompatibility and bioactivity, supporting cell adhesion across the entire 3D scaffold and offering a promising strategy for antimicrobial bone regeneration.^7^

Once the final design was approved by the surgical team, the implant was manufactured and delivered within a maximum of 3 weeks. In practice, production times were often shorter, ranging from 10 to 15 days in high-priority cases. This workflow allowed for timely coordination with the surgical schedule, including urgent oncologic procedures.

The cost of each prosthesis ranged between €7500 and €15,000, depending on size and complexity. Complexity was determined by factors such as the anatomical extent of the defect, the number of structural elements involved (eg, ribs, sternum, joints), and the need for dynamic components.

**Figure 1:**
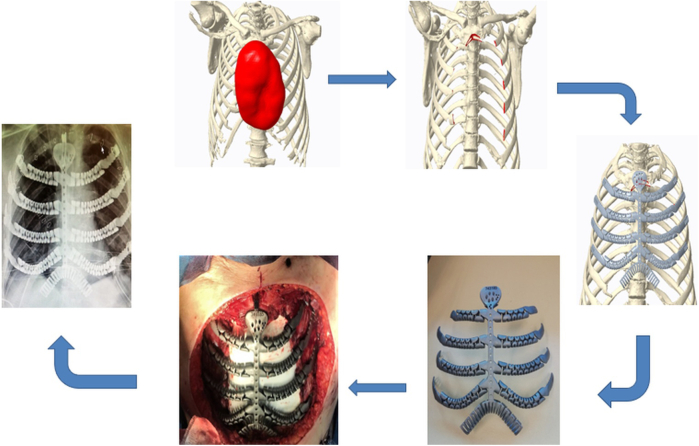
Redefining chest wall reconstruction with dynamic 3-dimensional implants: structure meets function.

The complete workflow from virtual planning to final postoperative outcome is illustrated in Figure 1. Variables analyzed included the date of implantation, surgical indication, patient age and sex, respiratory and cardiac comorbidities, type of implant, use of myoplasty, use of synthetic or biological meshes, operative time, length of hospital stay, postoperative complications, and follow-up duration (Table 1).

**Table 1:** Postoperative outcomes, complications, follow-up, and survival by center

Variables	CHUIMI	HURYC	H U Cruces	H SanPau/Mar	H Ribera	Total
Postoperative d (range)	5.5 (1-12)	11.6 (5-28)	9.8 (7-10)	13 (7-22)	2 (2)	8.38 (1-28)
Medical or surgical complications, n	1	1	1	3	0	6
Breakage or migration of prosthesis, n	1	0	0	0	0	1
Follow up, y (range)	3.5 (0.5-8)	1.25 (0.5-2)	1.6 (0.5-3)	0.5 (0.25-0.9)	1.5 (1-2)	2.5 (0.25-8)
Results, n						
Alive without prosthesis failure	18	9	8	8	3	46
Alive with prosthesis failure	0	0	0	0	0	0
Deceased	3	2	0	0	0	5

*CHUIMI*, Complejo Hospitalario Universitario Insular Materno Infantil; *HURYC*, Hospital Ramon y Cajal*; H U Cruces*, Hospital Universitario Cruces; *HSanPau/Mar*, Hospital de la Santa Creu i Sant Pau; *H Ribera*, Hospital Universitario de La Ribera.

Outcomes included hospital stay, postoperative complications, prosthesis-related events (such as fracture or migration), follow-up duration, and patient status at last follow-up. Exposure was defined as the implantation of a custom-made dynamic 3D-printed prosthesis.

All patients provided informed written consent both for the use of their data in this study and for the publication of such data. Statistical analyses were performed using STATA/IC 16.1 (StataCorp). Continuous variables were tested for normality using the Shapiro-Wilk test and for homoscedasticity using the Levene test. Normally distributed variables are reported as means and ranges; categorical variables are presented as absolute counts. This study was reported in accordance with the STrengthening the Reporting of OBservational studies in Epidemiology guidelines for observational studies.

## Results

Since 2016, a total of 51 customized 3D-printed prostheses have been implanted across 5 hospitals in Spain for oncologic (n = 35), traumatic/chest wall hernia (n = 11), infectious (n = 3), or winged scapula (n = 2) indications. All prostheses were designed by the ITC and manufactured in collaboration with Osteobionix. The median patient age was 52 years (range, 15-78 years), with a slightly greater proportion of female patients (n = 29) than male patients (n = 22). Preexisting comorbidities were uncommon, with 3 cases of respiratory disease and 1 case of cardiovascular disease.

**Figure 2:**
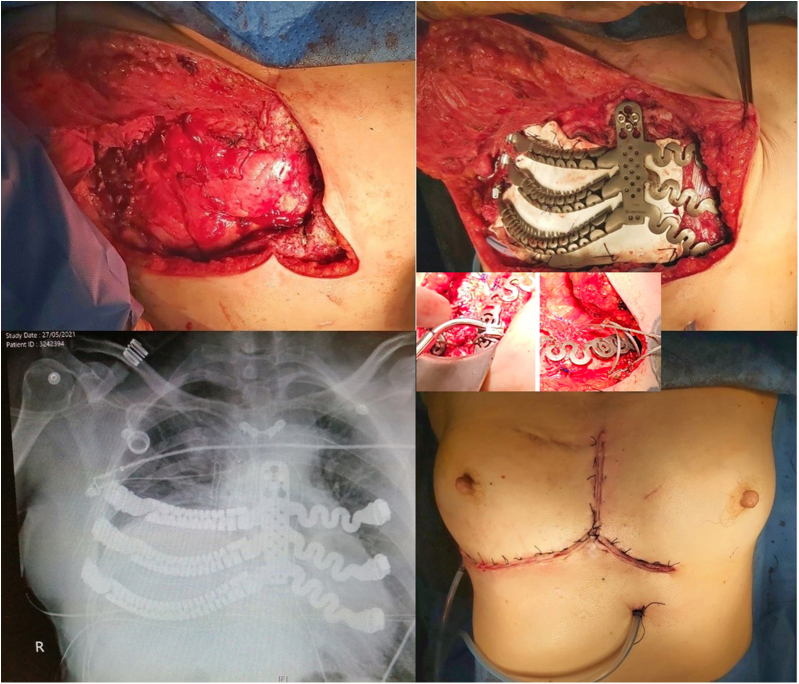
Intraoperative imaging of bilateral wall involvement with bilateral sternocostal prosthesis and postoperative radiograph.

In cases of sternocostal involvement or extensive costal, a prosthesis was designed to replicate the sternum—if resected—and costal arches using a dynamic tubular system. The rib ends were connected to the remaining native ribs using tensioned braided wires locked with a small padlock, whereas fixation to the sternum was achieved using custom self-locking screws (Figure 2).

**Figure 3:**
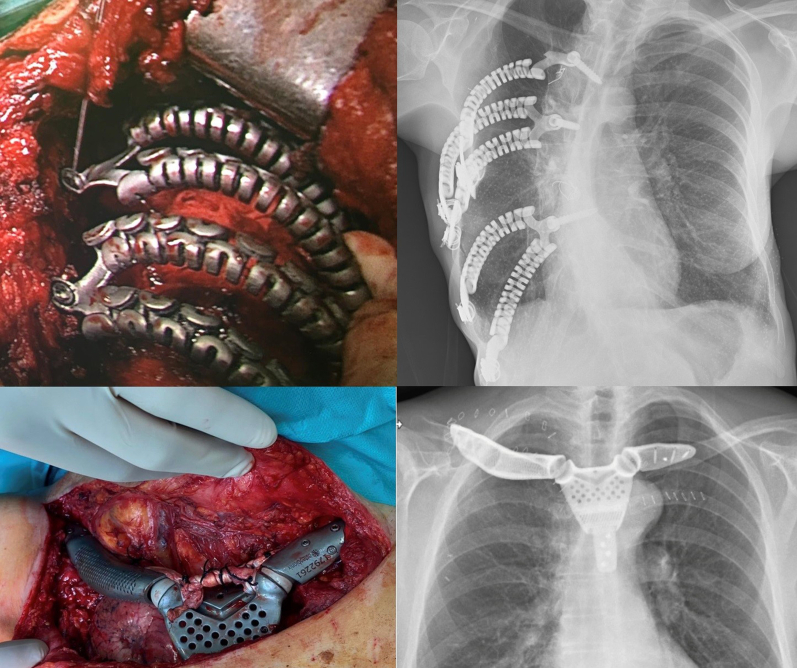
Intraoperative image of costovertebral and sternoclavicular prosthesis and postoperative radiograph.

In cases of costovertebral involvement, typically attributable to posterior tumors, fixation was achieved using a cannulated screw anchored into the vertebral body. One or 2 dynamic tubular branches extended from this point and were secured to the adjacent ribs using braided wire (Figure 3).

When the sternoclavicular joint was resected, it was reconstructed using a ball-and-socket prosthetic design. A polyether ether ketone ball was interposed between the clavicular and sternal prostheses, stabilized with a tendon graft from a tissue bank, and secured to both bones with self-locking screws (Figure 3).

**Figure 4:**
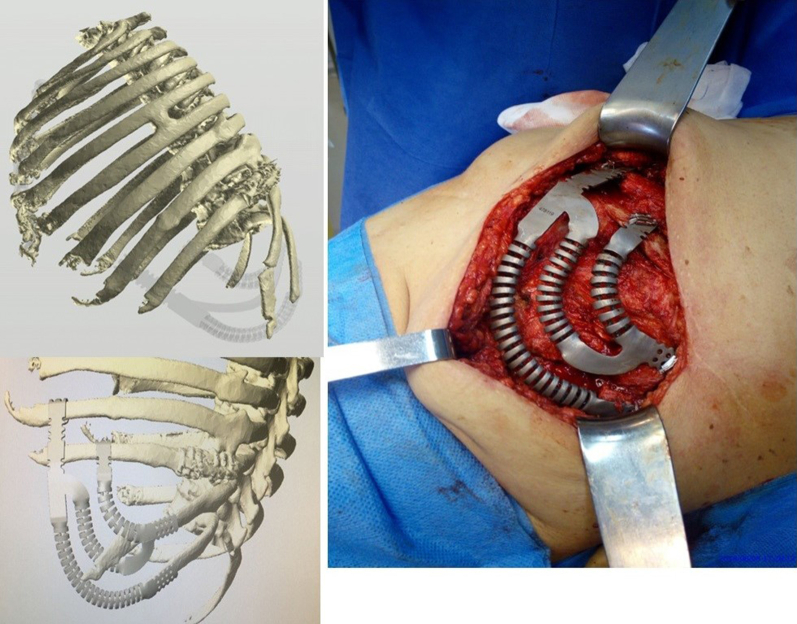
Three-dimensional preoperative reconstruction and intraoperative imaging with costal arch prosthesis.

Large posttraumatic chest wall hernias were reconstructed using the dynamic tubular system configured to mimic a costal arch. Additional branches extended to the ribs to cover the defect. Alternatively, the tubular system was shaped to serve as direct rib replacements (Figure 4).

**Figure 5:**
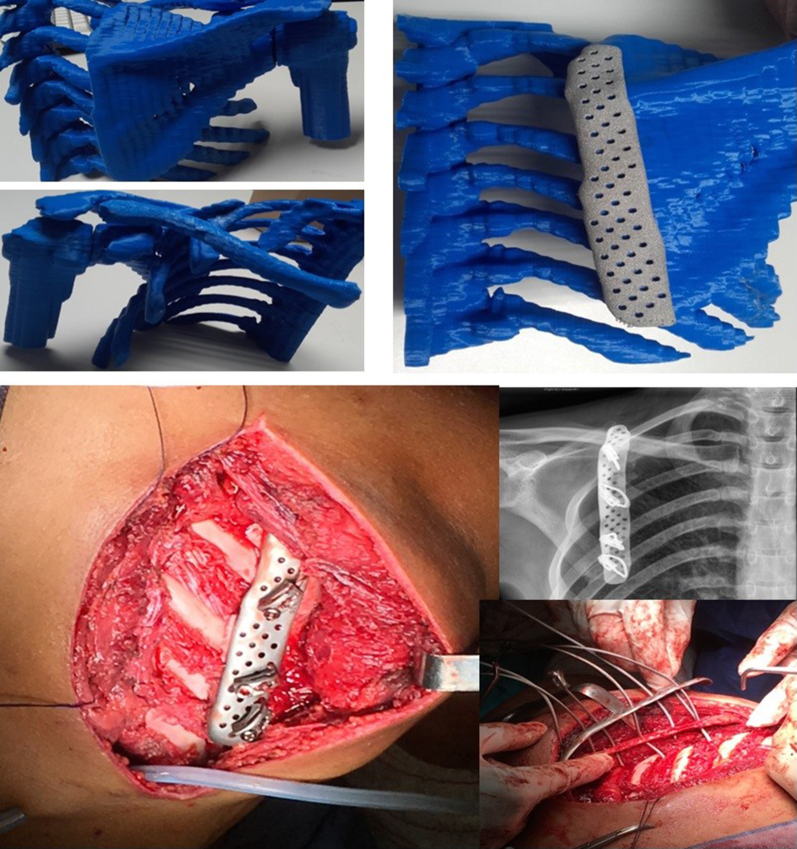
Preoperative model and intraoperative imaging of a winged scapula prosthesis and postoperative radiograph.

Winged scapula was treated with scapulothoracic arthrodesis. The scapula was fixed to the posterior rib cage at a 70° angle to allow for functional arm elevation above the horizontal plane. Given the fragility of the scapula—being a thin and flat bone—there is a risk of fracture during fixation. To address this, custom prostheses were designed to act as structural supports and to facilitate arthrodesis to the posterior chest wall. The implants included predrilled holes that allowed the scapula to be sutured to the posterior ribs using braided wire, while distributing mechanical stress through the prosthesis itself, thereby minimizing the risk of bone damage (Figure 5).

After fixation of the implant and closure of the soft tissues, all patients received submuscular Jackson-Pratt drains. Drain management was guided by clinical judgment and drain output. Patients were discharged with drains only when output was low and stable, and follow-up instructions were clearly defined.

Most procedures were complex and required additional reconstructive measures. Myoplasty was performed in 36 cases, whereas mesh reinforcement was used in 39, including biological meshes in 23 cases and synthetic meshes in 16. The median operative time was 270 minutes (range, 75-720 minutes), and postoperative hospital stays ranged from 2 to 28 days, depending on procedural complexity. A representative case showing real-time prosthetic movement during respiration is presented in Video 1, intended to illustrate the mechanical behavior of the implant in vivo.

A total of 6 prosthesis-related complications were documented. Two patients developed hematoma and infection, both of which were successfully managed with surgical revision without implant removal. One patient experienced venous thrombosis of the muscle flap, requiring additional coverage with a new flap. Respiratory failure occurred in one patient, necessitating prolonged mechanical ventilation. Another patient underwent revision surgery for postoperative bleeding, although the prosthesis was preserved. Notably, 1 prosthesis sustained fracture of the costal branches after a traffic accident, but surgical intervention was not required. An additional case of prosthesis migration was reported, without clinical consequences.

A total of 35 chest wall tumors requiring prosthetic reconstruction were identified across 4 centers in Spain and classified into 3 categories: primary chest wall tumors (n = 21), metastatic lesions from distant primaries (n = 7), and tumors with direct invasion from neighboring intrathoracic or breast malignancies (n = 7).

Among the primary tumors, chondrosarcoma was the most common histologic subtype (n = 6). In the metastatic group, chest wall involvement originated from advanced-stage carcinomas of the kidney, breast, prostate, and lung, with renal cell carcinoma being slightly more represented. All metastatic cases were confirmed as solitary lesions through rigorous staging workup and involved patients with preserved performance status. In these cases, complete surgical resection was performed as a salvage procedure, representing the only remaining therapeutic option. Tumors with direct chest wall invasion included those originating from the lung, thymus, and breast. Histologies in this group comprised squamous cell carcinoma of pulmonary origin, pleomorphic sarcoma, and type B2 thymoma. Neoadjuvant or adjuvant treatments were administered in selected cases, according to tumor histology, stage, and established international guidelines, and were adapted individually in multidisciplinary tumor boards.

Two patients presented with infectious conditions before prosthesis implantation. One had a chronic sternocutaneous fistula with osteolysis attributable to tuberculosis and underwent bilateral sternocostal reconstruction after resolution of their infection with antituberculosis therapy. The other presented with unilateral sternoclavicular joint destruction secondary to chronic infection and received a unilateral sternoclavicular prosthesis after complete eradication of the infection.

At a median follow-up of 2.5 years (range, 0.25-8 years), 46 patients remained alive and free of prosthesis failure. Five patients died, all because of recurrence of oncologic disease. No cases were lost to follow-up.

Notably, no cases of prosthesis failure occurred independently of patient death, underscoring the long-term durability of these implants. A detailed hospital-level breakdown is provided in Table 2 and Table E1.

**Table 2:** Patient and surgical characteristics across 5 centers

Variables	CHUIMI	HURYC	H U Cruces	HSanPau/Mar	H Ribera	Total
Cases, n	21	11	8	8	3	51
Type of pathology, n						
Neoplasm	12	9	6	8	0	35
Trauma-Thoracic wall hernia	5	2	1	0	3	11
Winged scapula	2	0	0	0	0	2
Infection	2	0	1	0	0	3
Middle age, y (range)	54 (15-78)	56 (25-76)	43 (30-68)	55 (26-77)	67 (57-73)	52 (15-78)
Sex, n						
Male	10	5	3	4	0	22
Female	11	6	5	4	3	29
Respiratory comorbidity, n	0	2	0	1	0	3
Heart comorbidity, n	1	0	0	0	0	0
Type of prosthesis, n						
Bilateral sternocostal	9	4	1	1	0	15
Unilateral sternocostal	3	3	4	5	0	15
Costal-Costal arch	3 + 3	3	1	0	3	13
Costovertebral	0	1	1	0	0	2
Sternoclavicular joint	1	0	1	2	0	4
Winged scapula	2	0	0	0	0	2
Myoplasty flap, n	14	7	5	8	0	36
Mesh, n						
Biological	4	6	5	8	0	23
Synthetic	10	5	0	0	1	16
Surgical time, min (range)	230 (120-360)	520 (260-720)	315 (200-480)	240 (180-300)	83 (75-90)	270 (75-720)

*CHUIMI*, Complejo Hospitalario Universitario Insular Materno Infantil; *HURYC*, Hospital Ramon y Cajal*; H U Cruces*, Hospital Universitario Cruces; *HSanPau/Mar*, Hospital de la Santa Creu i Sant Pau; *H Ribera*, Hospital Universitario de La Ribera.

## Discussion

This multicenter retrospective study suggests that custom-made dynamic 3D-printed prostheses represent a feasible and safe option for complex chest wall reconstruction. A total of 51 implants were performed across 5 centers in Spain for oncologic, infectious, and traumatic indications, including sternocostal, costovertebral, and sternoclavicular defects, as well as posttraumatic hernias and winged scapula. At a median follow-up of 2.5 years, 46 patients were alive with no evidence of prosthesis failure. All 5 deaths occurred in the oncologic subgroup and were related to disease progression.

Chest wall reconstruction in oncologic or traumatic settings remains surgically demanding and technically variable. Traditional approaches—such as mesh, cement, plates, or bars, often combined with muscle flaps—have proven effective for defect closure and organ protection, but their rigidity imposes significant limitations.^8^ These include the need for high surgical expertise, poor adaptability during implantation, risk of implant fracture, and pain associated with migration, often requiring removal.^9^ In contrast, dynamic implants are intended to better adapt to patient anatomy and movement, although further validation is needed.

The first reported case of a 3D-printed, patient-specific chest wall prosthesis was published by Turna and colleagues^2^ in 2014. This pioneering device consisted of a single rigid plate replacing the sternum and adjacent ribs, enabling accurate anatomical reconstruction but lacking flexibility and the ability to adapt to the dynamic behavior of costal cartilage. A slightly more advanced prosthesis—although still based on a static design—was introduced by Aranda and colleagues^3^ in 2015. Since then, most prostheses, whether serial or customized, have provided sufficient rigidity and protection of intrathoracic organs but often restrict physiological respiratory motion, which may predispose to early- or midterm implant loosening. Compared with traditional rigid devices—such as metal mesh, methyl methacrylate, or bars—and to newer rigid 3D-printed prostheses, these static systems may contribute to increased postoperative pulmonary complications and long-term restrictive ventilatory impairment.^10^^,^^11^ Many patients have subjectively described the sensation of “wearing a shell” that limits full chest expansion during breathing.

Since 2016, a novel class of custom-made dynamic prostheses developed by the ITC has been used in Spain, with selected cases previously reported by Aragón and Méndez,^12^ Moradiellos and colleagues,^13^ Cano and colleagues,^6^^,^^14^ and Vannucci and colleagues.^15^ These implants are considered “dynamic” as the result of their spring-like architecture—specifically, ribs articulated to a central sternal component—and are designed to approximate the structural behavior of costal cartilage. Postoperative imaging suggests that this design may contribute to maintaining the ventilatory behavior of the native chest wall, potentially preserving respiratory function.

The dynamic 3D-customized prosthesis described in this study addresses the previously mentioned limitations. It provides a tailored, site-specific solution—applicable to anterior chest wall, costovertebral, or sternoclavicular reconstructions. Its modular, spring-like tubular design also facilitates implantation and tolerates minor intraoperative deviations from preoperative computed tomography imaging, which rigid implants may noy accommodate as easily.^16^ The combination of dynamic 3D-customized implants and meticulous preoperative planning has enhanced procedural reproducibility across institutions, as demonstrated in our multicenter experience.

Fixation technique appears to be a critical determinant of surgical success when using dynamic prostheses. The choice of fixation method should be guided by both anatomical constraints and functional goals, balancing stability and mobility. Rigid fixation, such as with locking screws, may be preferable in central areas requiring structural support (eg, sternum, vertebral bodies), whereas more elastic fixation—such as braided wires—is better suited for ribs, minimizing the risk of fracture and preserving chest wall compliance. In selected cases, tendon grafts may offer an effective solution when joint articulation is required, such as in sternoclavicular reconstructions. These strategies highlight the need for individualized fixation planning in complex chest wall reconstruction.

No prosthesis-related complications were linked to patient age. Adverse events were infrequent and included 1 prosthesis fracture after high-impact trauma, 1 case of respiratory failure requiring prolonged ventilation, and 2 cases of hematoma or infection—none required implant removal. These outcomes appear favorable in light of previous reports, such as those by Losken and colleagues,^17^ who observed greater complication rates, infections, pneumonia, and longer hospital stays in patients treated with rigid prostheses versus primary closure.

As the design and manufacturing processes rely on globally available parametric computer-aided design software (CREO), medical-grade titanium alloys, and widely adopted additive manufacturing technologies such as EBM and selective laser melting, this approach could feasibly be replicated in other countries with access to qualified 3D-printing facilities, potentially expanding the availability of custom-made dynamic chest wall prostheses worldwide.

Beyond surgical feasibility, timely implant production is particularly relevant in oncologic cases, where delays in resection may affect prognosis. In our experience, close coordination between surgical teams and biomedical engineers enabled implant delivery within clinically acceptable time frames. Although our results suggest that this workflow is feasible, broader implementation will depend on local infrastructure and further validation in prospective settings.

This multicenter experience supports the technical feasibility and apparent safety of dynamic custom-made 3D-printed prostheses for complex chest wall reconstruction. However, due to the heterogeneity of indications, absence of a control group, and retrospective design, this study should be interpreted strictly as a proof-of-concept and surgical feasibility analysis. The lack of standardized assessments of functional outcomes—such as pulmonary function, pain, or quality of life—further limits any clinical extrapolation.

To address these limitations, we have launched a prospective, multicenter study (ClinicalTrials.gov NCT07018960) to evaluate long-term outcomes, including respiratory performance, patient-reported quality of life, and prosthesis durability. These data will be essential to refine the indications and assess the potential benefits of dynamic chest wall reconstruction.

## Conclusions

**Figure 6:**
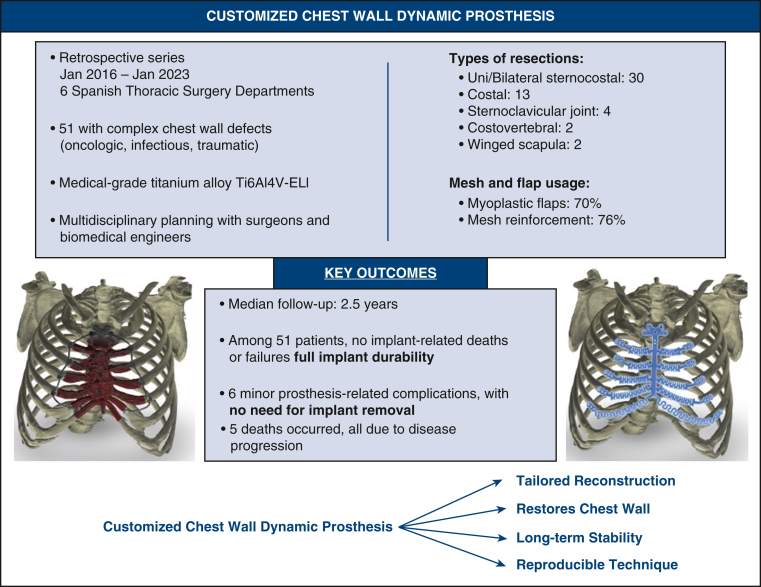
Study design, surgical indications, types of resections, materials used, and key outcomes. The customized dynamic prosthesis offers tailored reconstruction, restores chest wall stability and motion, and shows full implant durability at 2.5 years of follow-up. *Ti6Al4V-ELI*, Titanium alloy with aluminum and vanadium (extra low interstitials).

On the basis of our multicenter experience, we suggest that the use of dynamic 3D-customized prostheses for extensive chest wall reconstructions may be viable in selected cases, particularly those involving sternectomy, anterior defects, or joint resections (sternoclavicular or costovertebral). Their application is also appropriate in complex posttraumatic deformities—such as chest wall hernias and winged scapula—where conventional techniques may offer limited adaptability. A graphical abstract summarizing the key elements of this study—case distribution, surgical approach, and outcomes—is presented in Figure 6.

## Conflict of Interest Statement

The authors reported no conflicts of interest.

The *Journal* policy requires editors and reviewers to disclose conflicts of interest and to decline handling or reviewing manuscripts for which they may have a conflict of interest. The editors and reviewers of this article have no conflicts of interest.

## Figures and Tables

**Table E1:** Postoperative outcomes and follow-up summary by center

Variables	CHUIMI	HURYC	HU Cruces	H SanPau/Mar	H Ribera	Total
Postoperative d (range)	5.5 (1-12)	11.6 (5-28)	9.8 (7-10)	13 (7-22)	2 (2)	8.38 (1-28)
Medical or surgical complication, n	1	1	1	3	0	6
Breakage or migration prostheses, n	1	0	0	0	0	1
Follow up, y (range)	3.5 (0.5-8)	1.25 (0.5-2)	1.6 (0.5-3)	0.5 (0.25-0.9)	1.5 (1-2)	2.5 (0.25-8)
Results, n						
Alive without prosthesis failure	18	9	8	8	3	46
Alive with prosthesis failure	0	0	0	0	0	0
Deceased	3	2	0	0	0	5

*CHUIMI*, Complejo Hospitalario Universitario Insular Materno Infantil; *HURYC*, Hospital Ramon y Cajal*; H U Cruces*, Hospital Universitario Cruces; *HSanPau/Mar*, Hospital de la Santa Creu i Sant Pau; *H Ribera*, Hospital Universitario de La Ribera.

## Supplementary Data

Video 1 Dynamic radiograph showing breathing movements with the dynamic prosthesis in place. Video available at: https://www.jtcvs.org/article/S2666-2507(25)00375-X/fulltext.

## References

[1] Sanna S., Brandolini J., Pardolesi A. (2017). Materials and techniques in chest wall reconstruction: a review. J Vis Surg.

[2] Turna A., Kavakli K., Sapmaz E. (2014). Reconstruction with a patient-specific titanium implant after a wide anterior chest wall resection. Interact Cardiovasc Thorac Surg.

[3] Aranda J.L., Jiménez M.F., Rodríguez M., Varela G. (2015). Tridimensional titanium-printed custom-made prosthesis for sternocostal reconstruction. Eur J Cardiothorac Surg.

[4] Wen X., Gao S., Feng J., Li S., Gao R., Zhang G. (2018). Chest wall reconstruction with a customized titanium-alloy prosthesis fabricated by 3D printing and rapid prototyping. J Cardiothorac Surg.

[5] Simal I., García-Casillas M.A., Cerdá J.A. (2016). Three-dimensional custom-made titanium ribs for reconstruction of a large chest wall defect. European J Pediatr Surg Rep.

[6] Cano J.R., Escobar F.H., Alonso D.P., Rivero L.L. (2018). Reconstruction of the anterior chest wall with a 3-dimensionally printed biodynamic prosthesis. J Thorac Cardiovasc Surg.

[7] Rodriguez-Palomo A., Monopoli D., Afonso H., Izquierdo-Barba I., Vallet-Regí M. (2016). Surface zwitterionization of customized 3D Ti6Al4V scaffolds: a promising alternative to eradicate bone infection. J Mater Chem B.

[8] Bergovec M., Smolle M., Lindenmann J., Fediuk M., Leithner A., Smolle-Jüttner F.M. (2022). High complication rate with titanium plates for chest wall reconstruction following tumour resection. Eur J Cardiothorac Surg.

[9] Hazel K., Weyant M.J. (2015). Chest wall resection and reconstruction: management of complications. Thorac Surg Clin.

[10] Elahi L., Zellweger M., Abdelnour-Berchtold E. (2022). The size and sternal involvement of chest wall resections for malignant disease predict postoperative morbidity. Transl Cancer Res.

[11] Goldsmith I. (2022). Chest wall reconstruction with 3D printing: anatomical and functional considerations. Innovations (Phila).

[12] Aragón J., Méndez I.P. (2016). Dynamic 3D printed titanium copy prosthesis: a novel design for large chest wall resection and reconstruction. J Thorac Dis.

[13] Moradiellos J., Amor S., Córdoba M., Rocco G., Vidal M., Varela A. (2017). Functional chest wall reconstruction with a biomechanical three-dimensionally printed implant. Ann Thorac Surg.

[14] Cano J.R., Leung Shao M., Monopoli D. (2024). Giant reconstruction of right hemithorax with 3D custom dynamic prosthesis. Arch Bronconeumol.

[15] Vannucci J., Scarnecchia E., Potenza R., Ceccarelli S., Monopoli D., Puma F. (2020). Dynamic titanium prosthesis based on 3D-printed replica for chest wall resection and reconstruction. Transl Lung Cancer Res.

[16] Fra S., Muñoz-Molina G.M., Cano J.R. (2025). Innovative posterior chest wall reconstruction with vertebra-fixed custom-made prosthesis. J Thorac Cardiovasc Surg Tech.

[17] Losken A., Thourani V.H., Carlson G.W. (2004). A reconstructive algorithm for plastic surgery following extensive chest wall resection. Br J Plast Surg.

