# Supplementary figures and images for: Custom-made dynamic 3-dimensional−printed prostheses for chest wall reconstruction: A multicenter study
Source: JTCVS Tech. 2025 Sep 19;34:269–79. doi: 10.1016/j.xjtc.2025.09.010 (PMC12683048; doi:10.1016/j.xjtc.2025.09.010)

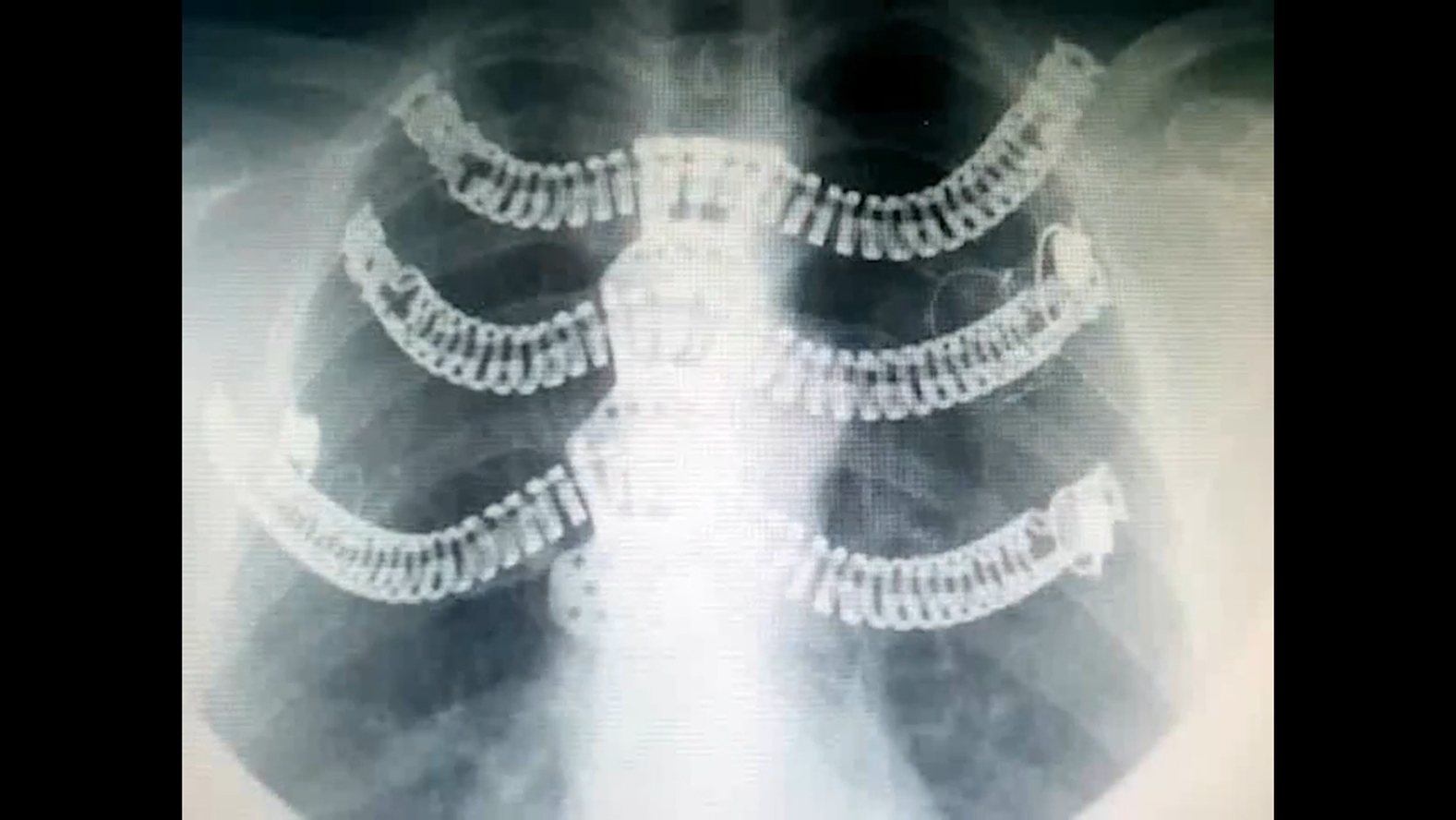

Supplement: Video 1 — Dynamic radiograph showing breathing movements with the dynamic prosthesis in place. Video available at: https://www.jtcvs.org/article/S2666-2507(25)00375-X/fulltext. [file fx2.jpg]
